# High Prevalence of *Schistosoma japonicum* and *Fasciola gigantica* in Bovines from Northern Samar, the Philippines

**DOI:** 10.1371/journal.pntd.0003108

**Published:** 2015-02-02

**Authors:** Catherine A. Gordon, Luz P. Acosta, Geoffrey N. Gobert, Mario Jiz, Remigio M. Olveda, Allen G. Ross, Darren J. Gray, Gail M. Williams, Donald Harn, Yuesheng Li, Donald P. McManus

**Affiliations:** 1 Molecular Parasitology Laboratory, Infectious Diseases Division, QIMR Berghofer Institute of Medical Research, Brisbane, Australia; 2 Infectious Disease Epidemiology Unit, School of Population Health, University of Queensland, Brisbane, Australia; 3 Department of Immunology, Research Institute of Tropical Medicine, Manila, Philippines; 4 Griffith Health Institute, Griffith University, Brisbane, Australia; 5 Research School of Population Health, the Australian National University, Canberra, Australia; 6 Discipline of Epidemiology and Biostatistics, School of Population Health, University of Queensland, Brisbane, Australia; 7 University of Georgia, College of Veterinary Medicine, Athens, Georgia, United States of America; Asahikawa Medical College, JAPAN

## Abstract

The cause of zoonotic schistosomiasis in the Philippines is *Schistosoma japonicum*, which infects up to 46 mammalian hosts, including humans and bovines. In China, water buffaloes have been identified as major reservoir hosts for schistosomiasis japonica, contributing up to 75% of human transmission. In the Philippines, water buffaloes (carabao; *Bubalus bubalis carabanesis*) have, historically, been considered unimportant reservoirs. We therefore revisited the possible role of bovines in schistosome transmission in the Philippines, using the recently described formalin-ethyl acetate sedimentation (FEA-SD) technique and a qPCR assay to examine fecal samples from 153 bovines (both carabao and cattle) from six barangays in Northern Samar. A high prevalence of *S. japonicum* was found using qPCR and FEA-SD in both cattle (87.50% and 77.08%, respectively) and carabao (80.00% and 55.24%, respectively). The average daily egg output for each bovine was calculated at 195,000. High prevalence and infection intensity of *F. gigantica* was also found in the bovines by qPCR and FEA-SD (95.33% and 96.00%, respectively). The identification of bovines as major reservoir hosts for *S. japonicum* transmission suggests that bovine treatment and/or vaccination, as one becomes available, should be included in any future control program that aims to reduce the disease burden due to schistosomiasis in the Philippines.

## Author Summary

Schistosomiasis japonica, a zoonosis of over 40 different mammalian species, is endemic to China, the Philippines and Indonesia. In China, water buffaloes have been shown to be major reservoir hosts, while in the Philippines, the smaller sub species (carabao) has been been considered unimportant in transmission, possibly due to the lack of sensitive copro-parasitological techniques employed. We used an exhaustive microscopic technique, the FEA-SD, and a sensitive qPCR assay on a cohort of bovines to assess their potential role in transmission in the Philippines. Both cattle and carabao were highly infected with *Schistosoma japonicum* and *Fasciola gigantic* and co-infection was common. The high prevalence and intensity of bovine infection with *S*. *japonicum* suggest their heavy involvement in human transmission and that future control programs should target these reservoirs to reduce human infection.

## Introduction

*Schistosoma japonicum*, the cause of zoonotic schistosomiasis, infects more than 40 species of wild and domestic animals (including bovines, pigs, horses and goats) [1], complicating control efforts. Mathematical modelling predicts that up to 75% of *S*. *japonicum* transmission to humans is attributable to bovines in the lake and marshland areas of China [2]. This is due to the fact that infected water buffaloes and cattle excrete daily up to 60 kg of stool per individual [3–5]. With such a large volume of feces excreted daily, the potential number of eggs excreted is similarly high. This contrasts with rodents which excrete approximately 1 g of feces per day and humans which produce around 250 g daily [6]. Water buffaloes habitually spend much of their time immersed in water bodies, such as rivers, lakes and water holes, into which they tend to defecate directly, so that if *Oncomelania hupensis* are present, the likelihood of transmission is high.

While extensive studies have been undertaken on reservoir hosts in China [4,5,7–10], there are limited reports on the zoonotic potential of schistosomiasis japonica in the Philippines. This is despite the 2.88 million carabao present in the Philippines [11] (*Bubalis bubalis carabenensis*), a smaller sub-species of the Chinese water buffalo. Previous reports from the Philippines had recorded only low *S*. *japonicum* prevalence in carabao, suggesting that these bovines play a limited role in transmission [12–15]. However, a study in Leyte, a province located in the Eastern Visayas region, reported a *S*. *japonicum* prevalence of 52% in carabao using a quantitative real time polymerase chain reaction (qPCR) method [16]. Much lower prevalence values were obtained using the Kato Katz (KK) method (4%), miracidial hatching test (MHT) (0%) and the Danish Bilharziasis Laboratory (DBL) technique (4%) [16], suggesting caution regarding the involvement of carabao in transmission of schistosomiasis japonica. The Leyte study also highlighted the need for a more sensitive copro-parasitological technique for comparison with the qPCR.

Accordingly, in a pilot study conducted in Western Samar, located in the Eastern Visayas region of the Philippines, we recorded a high prevalence of *S*. *japonicum* in carabao using a validated real-time PCR (qPCR) and a new copro-parasitological tool, the formalin-ethyl acetate sedimentation (FEA-SD) technique [17,18]. A much lower prevalence of *S*. *japonicum* was recorded for the same fecal samples using conventional PCR, the Kato-Katz technique and MHT [17,18].

Here we report on a larger study in Palapag, a municipality in the province of Northern Samar where we determined the prevalence of *S*. *japonicum* in cattle and carabao. We also determined the prevalence of *Fasciola gigantica* in these bovines and investigated whether there is any cross-protective effect between this trematode species and *S*. *japonicum*. Fascioliasis in animals is a chronic disease and causes anaemia, lethargy, weight loss and lower fertility [19,20]. *F*. *gigantica* is the main causative agent of fascioliasis in the Philippines where it is the leading cause of bovine morbidity and mortality [20].

## Materials and Methods

### Ethics

Informed written consent was received from all animal owners in the study area and ethical approval for the animal work was provided by the Ethics Committee of the Research Institute of Tropical Medicine and the QIMR Berghofer Medical Research Institute Animal Research Ethics Committee (P288). This study was performed in accordance with the recommendations of the Australian code of practice for the care and use of animals for scientific purposes, 2004.

### Study design

We carried out a cross-sectional survey (July-September 2011) in the municipality of Palapag, Northern Samar Province, the Philippines, to determine the level of *S*. *japonicum* and *F*. *gigantica* infection in animals using the FEA-SD and qPCR methods. Primary endpoints were bovine prevalence and intensity of infection; secondary end points were sensitivity and specificity of the FEA-SD and qPCR techniques.

### Study area

**Fig 1:**
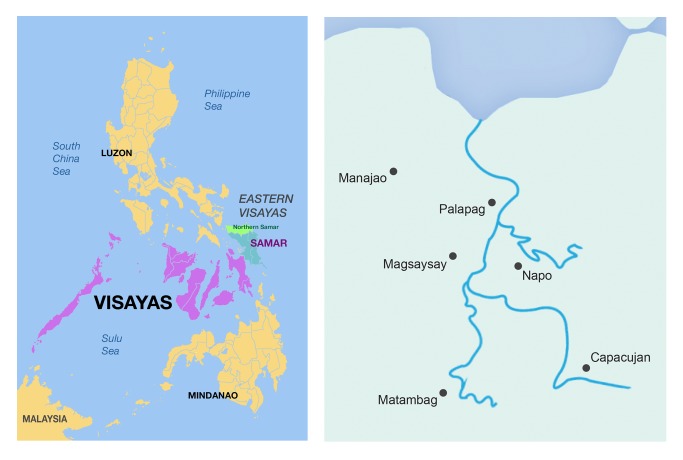
Map of the Philippines showing Northern Samar province highlighted red (Left). Map of the municipality of Palapag, showing barangay locations and rivers (Right).

The study was undertaken in six barangays; Napo, Capacujan, Matambag, Mabaras, Magsaysay and Manajao, all in the municipality of Palapag in Northern Samar Province (Fig. 1). Palapag was chosen due to the known endemicity of the municipality from government control records. No praziquantel treatment of bovines for schistosomiasis had occurred in the area prior to the study. A total of 153 bovine samples (48 cattle, 105carabao) were collected for analysis for *S*. *japonicum*; 150 bovine samples (45 cattle, 105 carabao) were analysed for the presence of *F*. *gigantica* infection. Age and gender of bovines was ascertained by use of a questionnaire given to the animal owners prior to fecal collection. Bovines surveyed in this study came from 112 different households, as determined by a household questionnaire, however there are many communal areas (rice fields and rivers) where bovines from different households are co-held.

### Study procedures

Bovine owners were requested to bring their animals to a central area on a stated day for faecal collection. Fecal samples from these animals were collected intra-rectally by a team of local veterinarians in plastic, re-sealable bags, and brought back to the local Palapag medical center. Once there, samples were stored for later qPCR analysis and for processing of the FEA-SD technique. For molecular analysis approximately 3 g of feces was placed into 5 ml tubes with sufficient 80% (v/v) ethanol to completely cover the sample. Tubes were stored at 4°C transported at room temperature to the QIMR Berghofer laboratory in Brisbane where DNA was extracted from the samples and qPCR performed. The FEA-SD was performed in Palapag and is described in detail below.

### FEA-SD

The published FEA-SD method [17,18] was used with some modifications. Briefly, 50 g of homogenized bovine stool was washed through a 60 nylon mesh (Tyler scale with a pore opening size of 250 μm) onto a 40 nylon mesh (Tyler scale with a pore opening size of 40 μm). The material retained on the 40 nylon mesh was washed into a 50 ml tube, allowed to sediment for 30 minutes, the supernatant was removed, the pellet re-suspended in 10% formalin (v/v) (mixed with tap water) and the sedimentation procedure repeated twice more. Ten ml of the final suspension was removed, placing 5 ml into two 15 ml tubes labeled A and B. Ten percent formalin (v/v) was added to each tube to take the volume to 8 ml and mixed thoroughly, after which 4 ml of 100% ethyl acetate (v/v) was added. Tubes were vortexed and centrifuged at 500 *g* for 10 minutes. The ethyl acetate layer was removed by gently rimming the tube with an applicator stick and the top layers poured off. The pellet was washed once with tap water and re-suspended to 5 ml with 10% formalin (v/v) and 5 ml of 10% potassium hydroxide (w/v) (KOH) added. The tubes were vortexed and the samples allowed to digest at 37°C for at least 6 hours before centrifugation at 900 *g* for ten minutes. The supernatant was removed, the pellet washed once with water and re-suspended in water, mixing gently with a pipette prior to microscopic examination for eggs.

The egg counts for *S*. *japonicum* were undertaken by Northern Samar regional staff and staff from the Research Institute of Tropical Medicine, Manila. Egg counts for *F*. *gigantica* were completed by one of the authors (CAG) at the QIMR Berghofer. *F*. *gigantica* eggs were measured to help distinguish from the morphologically similar eggs of paramphistomes (stomach fluke) which are also present in bovines in the Philippines [21]. Eggs over 160 μm in length were counted as *Paramphistomum* eggs, while those <160 μm were counted as *F*. *gigantica* eggs.

In the original protocol wet mounts of 200 μl aliquots were used for counting schistosome eggs [17,18]. In this study aliquots of 62.5 μl were used. Thus, egg counting for *S*. *japonicum* occurred in the following manner. Each tube (A and B) is equivalent to 5 g of feces, 10 g in total. A total of 500 μl suspension was read by microscopy for *S*. *japonicum* eggs (four slides prepared from each tube (8 slides in total); 62 μl per slide (using a small coverslip)), equivalent to 0.5 g of feces. To calculate eggs per gram of feces (EPG), the number of eggs counted on each slide per sample (both tube A and B) was multiplied by two.

For *F*. *gigantica* two slides from each tube were read by microscopy (4 slides, 200 μl aliquots each for a total of 800 μl suspension read). To calculate eggs per gram of feces (EPG), the total number of eggs counted on each slide was added together, divided by 8 and then multiplied by 10.

### DNA extraction

Genomic DNA was isolated from 200 mg of individual fecal samples stored in 80% ethanol using QIAamp mini stool kits (QIAGEN), following the manufacturers protocol. DNA concentration was determined using a NanoDrop 2000 (Thermo Scientific) with all samples diluted to 20 ng/μl for analysis.

### Real-time PCR

The *S*. *japonicum* qPCR assay was performed as previously described [11] and utilized primers which amplify a fragment of the NADH dehydrogenase I (*nad1*) mitochondrial gene [16,17,22]. The PCR conditions were as follows: 50°C initialization for 2 min, 95°C denaturation for 10 min, followed by 45 cycles of denaturation at 95°C for 15 sec, annealing at 60°C for 60 sec, extension at 72°C for 90 sec and a final dissociation phase at 60–95°C. The PCR was performed using a real-time thermocycler (Corbett RotorGene 6000) with melt curve analysis performed for each qPCR. The results were quantified as eggs per gram [EPG] (1–120 eggs per gram) using Ct (cycle threshold) scores as previously described [17]. Briefly, a series of egg seeding and dilution experiments were performed to relate egg numbers and amount of DNA to Ct scores, thereby creating a standard curve to calculate the number of eggs corresponding to a particular Ct score [17].

Primers for the *F*. *gigantica* qPCR were designed for this study, using online software primer plus and the IDT (integrated DNA technologies) oligonucleotide design tool, to amplify the NADH dehydrogenase I (*nad1*) mitochondrial gene: Forward primer (5ˈ-GAGATTTGCTGATTTGATGAAGTT-3ˈ); Reverse primer (5ˈ-CCAACAAATAAATCCCTCACC-3ˈ). The PCR conditions were as follows: 50°C initialization for 2 min, 95°C denaturation for 10 min, followed by 40 cycles of denaturation at 95°C for 30 sec, annealing at 55°C for 30 sec, extension at 72°C for 30 sec and a final extension at 72°C for 10 minutes. The PCR was performed using a conventional thermocycler standard (Corbett RotorGene 6000). The amplicon for the qPCR was sequenced to confirm the DNA target and a BLAST search run on genbank to confirm specificity [17]. Additionally, DNA from *F*. *hepatica*, as a closely related species, was used as a control for the qPCR with the *F*. *gigantica* primers, to demonstrate that no non-specific amplification occurred.

### Statistical analyses

Microsoft Excel and SAS software (SAS Institute, Cary, NC) were used for data analyses. A sample was considered positive if there was at least one *S*. *japonicum* or *F*. *gigantica* egg on any FEA-SD slide; or if a positive Ct score was seen by qPCR (Ct score greater than 22.0 was considered as negative, less than 21.99 was considered positive). Egg counts were transformed to eggs per gram and geometric mean intensity calculated by using the log-transformed egg counts. Confidence limits were calculated using standard formulae based on the binomial distribution (prevalence) and the lognormal distribution (infection intensity). Relative diagnostic sensitivity and specificity of FEA-SD were calculated using qPCR as the reference standard.

The bovine contamination index (BCI) was derived using the previously published formula [23] using data obtained with the FEA-SD technique.

BCI = [arithmetic mean of eggs per gram (epg) (of infected bovines)] x number of infected bovines] x 25, 000 (grams fecal weight)

In China it has been shown that bovines can excrete 25–50 kg of feces per day [3]. Here we chose 25 kg as a conservative estimate for the BCI calculation. BCI was calculated using data obtained from the FEA-SD technique which gave a more conservative epg than the qPCR, but also relied on egg visualization.

To account for clustering effects within barangays generalized estimating equations (GEE) were used to calculate p-values with barangay as a as a cluster effect.

Relative sensitivity and specificity of FEA-SD was calculated using qPCR as the reference standard.

## Results

### *Schistosoma japonicum*: Relative sensitivity and specificity of qPCR and FEA-SD

Using qPCR as the reference standard the FEA-SD had a sensitivity of 60.8% (95% CI 51.7–69.4) (Table 1) and specificity of 32.1% (95%CI 15.9–52.35).

**Table 1:** Prevalence and intensity (GMEPG*) of *S*. *japonicum* infections in bovines.

	Diagnostic Technique	N	No. Positive	Prevalence % (CI**)	GMEPG*
Cattle	qPCR	48	42	87.5 (77.8–97.2)	15.4 (9.8–24.0)
	FEA-SD	48	37	77.1 (64.8–89.4)	8.3 (6.4–10.7)
Carabao	qPCR	105	84	79.1 (71.1–87.0)	14.1 (11.1–17.9)
	FEA-SD	105	58	55.2 (45.6–64.9)	4.7 (4.0–5.4)
Bovines***	qPCR	153	126	81.7 (75.5–87.9)	14.5 (11.7–18.0)
	FEA-SD	153	95	62.1 (54.3–69.9)	5.9 (5.1–6.8)

* Geometric Eggs per Gram of feces in infected

**Confidence Interval

***Carabao and cattle data combined

### *Schistosoma japonicum*: Prevalence and intensity of infection

The prevalence of *S*. *japonicum* in all 153 bovines (carabao and cattle combined) determined by qPCR and FEA-SD was high–81.70% (95% CI 75.5–87.9) and 62.09% (95%CI 54.3–69.9) respectively (Table 1). The infection intensity for bovines by qPCR (GMEPG 14.5) was also higher than by the FEA-SD technique (GMEPG 5.9) (Table 1). The infection intensity was significantly (P = 0.0001) higher in cattle (GMEPG 8.3 (95%CI 6.4–10.7) than carabao (GMEPG 4.7 (95%CI 4.0–5.4) (Table 1). Barangay prevalence ranged from 55.56% (95% CI 15.0–96.1)– 100% for qPCR and 53.3% (95% CI 24.7–81.9)– 80.0% (95% CI 67.0–93.0) for FEA-SD. Manajao had the highest prevalence by qPCR, while Napo had the highest prevalence by FEA-SD (Table 2). The highest infection intensity by qPCR was in Mabaras (GMEPG 39.6 (95% CI 11.0–142.3)); although by FEA-SD it was in Matambag (GMEPG 6.9 (95% CI 5.4–8.7) (Table 2).

**Table 2:** Prevalence and intensity (GMEPG) of *S*. *japonicum* infections in bovines (carabao and cattle data combined) by gender, age and barangay.

		qPCR			FEA-SD		
	N	No. positive	Prevalence % (CI*)	GMEPG**	No. positive	Prevalence % (CI*)	GMEPG**
Total examined	153	125	81.7 (75.5–87.9)	14.5 (11.7–18.0)	95	62.1 (54.3–69.9)	5.9 (5.1–6.8)
Gender							
Male	19	15	79.0 (58.8–99.1)	16.1 (8.4–30.8)	11	57. 9 (33.5–82.3)	4.8 (3.3–6.9)
Female	134	110	82.1 (75.5–88.7)	14.3 (11.4–18.0)	84	63.0 (54.4–71.0)	6.0 (5.1–7.0)
Age Group (yrs)							
2 and under	36	30	83.3 (70.5–96.1)	15.1 (9.9.3–24.6)	36	55.6 (38.5–72.6)	6.4 (4.9–8.5)
Over 2	117	95	81.2 (74.0–88.4)	14.4 (11.3–18.3)	54	64.1 (55.3–72.9)	5.7 (4.8–6.8)
Barangay							
Capacujan	30	24	80.0 (64.8–95.2)	10.5 (6.6–16.6)	30	60.0 (41.439–78.6)	6.2 (3.8–10.3)
Napo	40	33	82.5 (70.2–94.8)	19.1 (12.5–29.1)	40	80.0 (67.0–93.0)	5.7 (4.4–7.2)
Matambag	30	20	66.7 (48.8–84.6)	8.9 (4.6–17.1)	30	50.0 (31.0–69.0)	6.9 (5.4–8.7)
Mabaras	9	5	55.6 (15.0–96.1)	39.6 (11.0–142.3)	9	66.7 (28.2–100)	4.8 (2.5–9.3)
Manajao	15	15	100 (N.A)	13.8 (7.8–24.3)	15	53.3 (24.7–81.9)	6.6 (4.2–10.4)
Magsaysay	29	28	96.6 (89.5–100)	17.0 (10.8–26.9)	29	55.2 (35.9–74.4)	5.1 (3.5–7.3)

*95% CI

** Geometric Eggs per Gram of feces in infected

The bovine contamination index (BCI) was higher in cattle (285, 000 eggs per day per animal) than in carabao (137, 500 eggs per day per animal), and gave an average of 195, 000 eggs per day when all bovines were considered together (Table 3).

**Table 3:** Bovine Contamination Index (BCI)* calculated using the FEA-SD data.

	Arithmetic Mean EPG	Number Infected	BCI Overall	BCI per bovine
Bovines**	7.8	95	18525000	195000
Carabao	5.5	58	7975000	137500
Cattle	11.40	37	10545000	285000

*Calculated using 25 kg as the average daily fecal output.

**Carabao and cattle data combined

### *Fasciola gigantica*: Sensitivity and specificity of qPCR and FEA-SD

A total of 150 bovine samples were examined for the presence *F*. *gigantica* eggs using the qPCR and FEA-SD techniques. The qPCR technique was used as the relative reference standard. Sensitivity of the FEA-SD was 96.5% (95%CI 91.1–98.5) and specificity was 14.3% (95%CI 0.42–64.1).

### *Fasciola gigantica*: Prevalence and intensity of infection

Both the FEA-SD and qPCR techniques established a high prevalence of *F*. *gigantica* in bovines in the study area (Table 4).

**Table 4:** Prevalence and intensity (GMEPG) of *F*. *gigantica* in bovines.

	Diagnostic Technique	N	No. positive	Prevalence (%)*	GMEPG**
Cattle	qPCR	45	42	93.3 (85.8–100)	N/A
	FEA-SD	45	44	97.8 (93.3–100)	174.0 (128.2–236.3)
Carabao	qPCR	105	101	96.2 (92.5–99.9)	N/A
	FEA-SD	105	100	95.2 (91.1–99.4)	38.5 (30.9–48.1)
Bovines***	qPCR	150	143	95.3 (91.9–98.8)	N/A
	FEA-SD	150	144	96.0 (92.8–99.2)	61.1 (49.4–74.5)

*95% Confidence Interval

** Geometric Eggs per Gram of feces in infected

***Carabao and cattle data combined.

Infection intensity was calculated using the FEA-SD data; and cattle (GMEPG 174.0 (95% CI 128.2–236.3) had a significantly (P = 0.0001) higher infection intensity than carabao (GMEPG 38.5 (95% CI 30.9–48.1)) (Table 4). Female bovines had a significantly (p = 0.0001) higher infection intensity (GMEPG 68.8 (95%CI 55.4–85.4) than male bovines (GMEPG 26.5 (95%CI 13.1–53.4)). The *F*. *gigantica* prevalence was high across all barangays with Mabaras having 100% prevalence by both diagnostic techniques (Table 5). There were no significant differences between age groups or barangay for infection intensity.

**Table 5:** Prevalence and intensity (GMEPG) of *F*. *gigantica* in bovines (carabao and cattle data combined) by gender, age and barangay.

		qPCR		FEA-SD		
	N	No. positive	Prevalence % (CI*)	No. positive	Prevalence % (CI*)	GMEPG**
Total examined	150	143	95.3 (91.9–98.8)	144	96.0 (92.8–99.2)	61.1 (49.4–75.5)
Gender						
Male	19	17	89.5 (74.3–100)	18	94.7 (83.7–100)	26.5 (13.1–53.4)
Female	131	126	96.2 (92.9–99.5)	126	96.2 (92.9–99.5)	68.8 (55.4–85.4)
Age Group (yrs)						
2 and under	36	31	86.1 (74.2–98.0)	31	86.1 (74.2–98.0)	47.4 (26.9–83.3)
Over 2	114	112	98.3 (95.8–100)	113	99.1 (97.4–100)	65.46 (52.3–82.0)
Barangay						
Capacujan	30	27	90.0 (78.6–100)	27	90.0 (78.6–100)	69.9 (39.3–124.3)
Napo	38	36	94.7 (87.3–100)	37	97.4 (92.0–100)	63.0 (37.0–107.1)
Matambag	30	30	100 (N/A)	29	96.7 (89.9–100)	43.6 (30.0–63.5)
Mabaras	9	9	100 (N/A)	9	100 (N/A)	81.2 (32.9–199.9)
Manajao	15	14	93.3 (79.0–100)	14	93.3 (79.0–100)	65.4 (37.6–113.7)
Magsaysay	28	27	96.4 (89.1–100)	28	100 (N/A)	64.3 (41.2–100.4)

*95% Confidence Interval

** Geometric Eggs per Gram of feces in infected

**NB**: 3 samples are missing from analysis as there was not enough material to repeat for the fasciola work.

### *Schistosoma japonicum* and *Fasciola gigantica*: Co-infections

The FEA-SD data were used to determine the prevalence and infection intensity of co-infections of *S*. *japonicum* and *F*. *gigantica* (Table 6). Of 150 bovines examined, 90 were co-infected with both *S*. *japonicum* and *F*. *gigantica* (34 cattle, 56 carabao). The infection intensity of animals infected with only *F*. *gigantica* or only *S*. *japonicum* was similar to the corresponding infection intensities of the co-infected animals (Table 6).

**Table 6:** Prevalence and GMEPG of co-infections of *S*. *japonicum* and *F*. *gigantica* in bovines using the FEA-SD technique.

	N	Prevalence % (CI*)	GMEPG**	GMEPG**
			(*F*. *gigantica*)	(*S*. *japonicum*)
Uninfected	3	2.7 (0–5.3)	N/A	N/A
*S*. *japonicum* and *F*. *gigantica*	90	60.0 (52.1–67.9)	67.0 (50.5–89.0)	5.8 (5.0–6.7)
*F*. *gigantica* only	54	36.0 (28.2–43.8)	52.3 (38.1–71.7)	N/A
S. japonicum only	3	2.7 (0–5.3)	N/A	7.3 (1.8–28.9)

*95% Confidence Interval

** Geometric Eggs per Gram of feces in infected

## Discussion

Both *F*. *gigantica* and *S*. *japonicum* were present in the great majority of carabao and cattle in Palapag (Tables 1, 4). The average BCI, calculated for *S*. *japonicum* using the FEA-SD data for all bovines showed that, on average, 195,000 eggs are released by each animal into the environment daily. The number calculated for cattle was higher, supporting previous studies indicating that cattle are more susceptible than the native carabao to infection with *S*. *japonicum* [24,25]. The average BCI calculated was higher than that calculated in our pilot investigation in Western Samar [17]. The current study was performed on a much larger scale with up to 153 animals being examined, thereby supporting the evidence of high prevalence and intensity of *S*. *japonicum* in another area of Samar.

Using qPCR as the reference standard, the FEA-SD was found to have moderate sensitivity and low specificity. This may have been due to the minor change in the published protocol for the microscopy following the FEA-SD procedure, resulting in a lower volume of fecal material read. In future, additional training should be undertaken to make sure that all protocols are strictly adhered to. Our previous pilot study [17] using the same techniques (FEA-SD and qPCR) found a higher sensitivity, 97.5% (95% CI 86.8–99.9), and specificity, 50.0% (95% CI 6.8–93.2). The low specificity in the pilot study was due to the small sample size (n = 44) as well as the low number of negative samples. Similarly, specificity of FEA-SD for *F*. *gigantica* was low due to the small numbers of samples that were negative, resulting in an unbalanced table. The majority of samples were positive by both techniques (n = 138).

Intensity of infection for both *F*. *gigantica* and *S*. *japonicum* was significantly higher in cattle compared with carabao. This observation is in agreement with the published literature which indicates that cattle are more susceptible to infection than the native carabao [1,25,26]. In experimental infections of cattle and water buffaloes in China, the average prepatency period for cattle (36.3±1.2) was shown to be less than that for water buffalo (42.0±1.7 days), and worm establishment is ten times greater in cattle than water buffalo [1]. Another study described the infection of six buffalo and six cattle, sacrificed after 7 weeks and compared the number of worms recovered, worm length, the hepatic granuloma response and the overall immune response [25]. It was found that more worms were recovered from cattle (29.7%) than buffalo (2.9%) and there were more worm pairs. Worms were also longer in cattle and there was evidence of a stronger immune response in the cattle when examining inflammatory cells and enlargement of the liver [25]. Despite the higher susceptibility of cattle to infection, the habitat of the water buffalo is more conducive to these bovines becoming infected with *S*. *japonicum* and transmitting the parasite.

The island of Samar is one of 10 administrative regions known to be endemic for schistosomiasis [27–29]. A pilot study conducted in Western Samar determined prevalences of 30.77%, 75% and 92.31% by KK, conventional PCR and real-time PCR respectively [17], rates much higher than previously reported. Human prevalence in Palapag is similarly high with an average prevalence of 22.9% and a GMEPG of 11.5 by KK, and 90.2%, GMPEG of 36.6 [17]. The high prevalence in Palapag is evident despite mass drug administration (MDA) undertaken there over the last five years [30,31], indicating that the program is not substantially reducing human infections so that other measures need to be considered. This feature makes the identification of a major disease reservoir even more pertinent.

Counting of eggs for *F*. *gigantica* and *S*. *japonicum*, following the FEA-SD technique, was performed separately, due to the morphological similarity between paramphistome eggs, which were found in nearly all bovines examined, and those of *F*. *gigantica*. Paramphistome eggs (160 μm x 90 μm) are slightly larger than those of *F*. *gigantica* (130–145 μm x 70–90 μm) necessitating the measurement of eggs to help differentiate between the two species.

However, both species are very similar in morphology and variations in the size of *Fasciola sp*. eggs have been shown to depend on different variables, such as the host species infected, with the resulting size variations showing overlap with the size of *Paramphistomum sp*. eggs [32]. It is therefore possible that some mis-identification occurred between the two species, if sizes were noted in the overlapping range. Thus, definitive identification between the two species morphologically and based on size is problematic, which makes molecular diagnosis an even more important tool. The qPCR we developed is absolutely specific for *F*. *gigantica*, as confirmed by sequencing products and BLAST searches using NCBI Genbank. DNA from the closely related species, *F*. *hepatica*, was used as a negative control to test the primers, with no amplification noted, again emphasising the specificity of the test.

The presence of fascioliasis in the Philippines has been documented previously in bovines and humans [20,33]. Transmission to humans is present, although occurs rarely due to dietary preferences. Consumption of raw water vegetables are the main source of human fascioliasis. In Mindanao a series of measures to prevent animal fascioliasis were taken, including building enclosures away from rice fields for bovines when they were not working, as well as storing and drying bovine feces thoroughly before using as fertilizer, all of which resulted in a decrease in animal fascioliasis and an increase in animal weights [20]. While the presence of schistosomiasis in these animals was not reported, application of these methods might also prove effective in decreasing animal schistosomiasis and therefore transmission to humans.

It has previously been shown that infection with *F*. *hepatica* may confer some resistance to infection with *S*. *mansoni* and vice versa [34–40], although similar studies have not been undertaken with *S*. *japonicum* and *F*. *gigantica*. Here we demonstrated a high level of co-infection with 60.00% of bovines harbouring both species (Table 6). The intensity of infection did not change significantly in animals infected with one or both species. The GMEPG for *S*. *japonicum* was slightly higher in animals infected only with *S*. *japonicum*, compared to those infected with both species. However, only three animals harboured *S*. *japonicum* alone, so this result may not represent the complete picture (Table 6). Conversely, the GMEPG for *F*. *gigantica* was higher in co-infected animals than in bovines infected only with *F*. *gigantica* (Table 6). Overall, these data tend to argue against the presence of any cross-protective phenomenon although definitive proof would require experimental studies involving single and co-infections of bovines with *S*. *japonicum* and *F*. *gigantica* so as to assess any effect on the intensity of infection, and to examine and compare immune response in the single and co-infected animals.

The differences in infection intensity obtained by the qPCR and FEA-SD techniques for *S*. *japonicum* may be attributed to the method whereby the EPG is calculated for the qPCR technique. The qPCR relies on approximations of how much DNA is in an egg and how it corresponds to a Ct score, which may result in an over or under estimation of infection intensity, and is therefore a semi-quantitative measure. Additionally, different extraction efficiencies can vary with different samples, resulting in variations in the total DNA isolated. As well, different samples may contain elevated levels of inhibitory components that impact the integrity of the qPCR. In contrast, the FEA-SD is fully quantitative as it relies on a physical count of eggs, rather than some of the approximations characteristic of the qPCR technique. Ideally the entire volume of sieved and digested feces would be read by mircoscopists which may result in a higher EPG. Due to constraints of time and cost we elected to do four slides per tube in this study.

## Conclusions

Historically, bovines have generally been considered unimportant in transmission of *S*. *japonicum* in the Philippines due to the low prevalence reported, or the inconsistencies in data obtained using coproparasitological and other diagnostic techniques [16,41,42]. Use of the FEA-SD technique results in the greatly improved visibility of eggs, and qPCR provides a more accurate appraisal of the role of bovines in schistosomiasis transmission.

The high prevalence and intensity of *S*. *japonicum* we report in bovines confirms the results of our earlier pilot study in Samar [15] and, as for China [3–7], suggests these animals play a major role in human transmission in the Philippines. Accordingly, an integrated approach to control using interventions that include bovine chemotherapy and/or vaccination should be considered to reduce the burden of schistosomiasis in the Philippines as has been advocated for the Chinese setting [43–45].
